# An exome-wide study of renal operational tolerance

**DOI:** 10.3389/fmed.2022.976248

**Published:** 2023-05-17

**Authors:** Annick Massart, Richard Danger, Catharina Olsen, Mary J. Emond, Ondrej Viklicky, Valérie Jacquemin, Julie Soblet, Sarah Duerinckx, Didier Croes, Camille Perazzolo, Petra Hruba, Dorien Daneels, Ben Caljon, Mehmet Sukru Sever, Julio Pascual, Marius Miglinas, the Renal Tolerance Investigators, Maria Aguilar Rodríguez, Isabelle Pirson, Lidia Ghisdal, Guillaume Smits, Magali Giral, Daniel Abramowicz, Marc Abramowicz, Sophie Brouard

**Affiliations:** ^1^Human Genetics Unit, Institut de Recherche Interdisciplinaire en Biologie Humaine et Moléculaire (IRIBHM), Université Libre de Bruxelles (ULB), Brussels, Belgium; ^2^Interuniversity Institute of Bioinformatics in Brussels (IB2), Université Libre de Bruxelles - Vrije Universiteit Brussel (ULB-VUB), Brussels, Belgium; ^3^Department of Nephrology, Antwerp University Hospital and Laboratory of Experimental Medicine, University of Antwerp, Antwerp, Belgium; ^4^CHU Nantes, Nantes Université, INSERM, Center for Research in Transplantation and Translational Immunology, CR2TI, UMR 1064, ITUN, Nantes, France; ^5^Brussels Interuniversity Genomics High Throughput Core (BRIGHTcore), VUB-ULB, Brussels, Belgium; ^6^Center for Medical Genetics, Reproduction and Genetics, Reproduction Genetics and Regenerative Medicine, Vrije Universiteit Brussel, UZ Brussel, Brussels, Belgium; ^7^Department of Biostatistics, University of Washington, Seattle, WA, United States; ^8^Transplant Laboratory, Institute for Clinical and Experimental Medicine, Prague, Czechia; ^9^Department of Genetics, Hôpital Erasme, ULB Center of Human Genetics, Université Libre de Bruxelles, Brussels, Belgium; ^10^Department of Genetics, Hôpital Universitaire des Enfants Reine Fabiola, ULB Center of Human Genetics, Université Libre de Bruxelles, Brussels, Belgium; ^11^Center for Human Genetics, Clinique Universitaires Saint Luc, Brussels, Belgium; ^12^Istanbul Tip Fakültesi, Istanbul School of Medicine, Internal Medicine, Nephrology, Istanbul, Türkiye; ^13^Department of Nephrology, Hospital del Mar, Institute Mar for Medical Research, Barcelona, Spain; ^14^Department of Nephrology, Hospital Universitario 12 de Octubre, Madrid, Spain; ^15^Nephrology Center, Santaros Klinikos, Medical Faculty, Vilnius University, Vilnius, Lithuania; ^16^Department of Nephrology, Hospital Centre EpiCURA, Baudour, Belgium; ^17^CHU Nantes, Centre d'Investigation Clinique en Biothérapie, Centre de Ressources Biologiques (CRB), Nantes, France; ^18^LabEx IGO “Immunotherapy, Graft, Oncology”, Nantes, France; ^19^Department of Genetic Medicine and Development, Faculty of Medicine, Université de Geneve, Geneva, Switzerland

**Keywords:** exome sequencing, renal transplantation, operational tolerance, NGAL, *LCN2*, *Homer2*, *IQCH*, primary cilium

## Abstract

**Background:**

Renal operational tolerance is a rare and beneficial state of prolonged renal allograft function in the absence of immunosuppression. The underlying mechanisms are unknown. We hypothesized that tolerance might be driven by inherited protein coding genetic variants with large effect, at least in some patients.

**Methods:**

We set up a European survey of over 218,000 renal transplant recipients and collected DNAs from 40 transplant recipients who maintained good allograft function without immunosuppression for at least 1 year. We performed an exome-wide association study comparing the distribution of moderate to high impact variants in 36 tolerant patients, selected for genetic homogeneity using principal component analysis, and 192 controls, using an optimal sequence-kernel association test adjusted for small samples.

**Results:**

We identified rare variants of *HOMER2* (3/36, FDR 0.0387), *IQCH* (5/36, FDR 0.0362), and *LCN2* (3/36, FDR 0.102) in 10 tolerant patients *vs*. 0 controls. One patient carried a variant in both *HOMER2* and *LCN2*. Furthermore, the three genes showed an identical variant in two patients each. The three genes are expressed at the primary cilium, a key structure in immune responses.

**Conclusion:**

Rare protein coding variants are associated with operational tolerance in a sizable portion of patients. Our findings have important implications for a better understanding of immune tolerance in transplantation and other fields of medicine.

ClinicalTrials.gov, identifier: NCT05124444.

## Introduction

Renal transplantation is considered the best treatment option for end-stage renal disease. However, graft survival comes at the price of life-long immunosuppression that may cause cardiovascular disease, cancer and infection. Exceptional patients will however maintain long-lasting allograft function, in spite of complete discontinuation of immunosuppression (1–3). The absence of destructive immunological response toward the allograft, contrasting with global immunocompetence and a normal response against other immunological challenges is referred to as 'operational tolerance'. Renal operational tolerance is associated with prolonged allograft and patient survival (1, 2, 4, 5). The discontinuation of immunosuppression in tolerant patients generally results from patient non-compliance or from medical decisions grounded on a life-threatening infection or cancer. However, under current transplantation protocols, and despite non-adherence being so common (6) spontaneous tolerance is very rarely observed. In a recent European survey, clinicians identified tolerance in 3/10,000 transplanted patients (2). Because non-compliance is usually undisclosed and in the absence of reliable biomarkers, most tolerant kidney recipients are discovered by chance, suggesting that the incidence of tolerance may be underestimated. Inducing renal tolerance in human recipients is possible through strategies of combined kidney and bone marrow transplantation, resulting in transient chimerism (7). Unfortunately, these strategies are challenging and far from routine clinical implementation. Thus, a better understanding of the molecular pathways and mechanisms involved in spontaneous tolerance might help design innovative tolerance induction protocols and medications.

Most studies of renal operational tolerance to date aimed at identifying reliable biomarkers for the detection of patients with reduced immunosuppression needs or for monitoring tolerance induction protocols [for review, see (3)]. Such studies included antigen-specific functional assays, circulating cell immuno-phenotyping, transcriptomic analyses and mechanistic studies. All together they showed that operational tolerance relies on the expansion of diverse alloreactive immune cells with inhibitory phenotypes and suppressive properties that maintain graft protection against inflammation injury. It remains unclear however whether the changes observed were causes or consequences of tolerance and currently, nothing explains why a kidney recipient initially receiving standard immunosuppression will eventually develop a form of immunoquiescence toward an alloreactive graft.

Because various extreme phenotypes are enriched in trait-causing alleles (8–14), we hypothesized that some tolerant patients may carry inherited, high impact gene variants allowing tolerance to develop. We reasoned that even Mendelian or near-Mendelian inheritance of tolerance would go unnoticed, because the phenotype is only revealed by transplantation, which is never performed experimentally in asymptomatic relatives of transplanted patients. As most high impact variants are found in protein-coding sequences of the genome (15), we sampled one of the world's largest DNA collections of tolerant patients (*n* = 40) and performed an exome-wide association study.

## Materials and methods

### Recruitment and data analysis

Patients were recruited from August 22, 2015 through October 29, 2019 and originated from transplantation centers in 12 different European countries (Austria, Belgium, Czech Republic, France, Germany, Holland, Italy, Lithuania, Romania, Spain, Switzerland, Turkey). Data analysis was performed from February 7, 2018 until September 29, 2021. The study protocol was approved at Erasme-ULB Ethics Committee under the refence 2015/379) and all participants signed a written informed consent form approved by their local institutional review board. This paper adheres to the Helsinki Declaration of 1975, as revised in 2013. The trial was registered in ClinicalTrials.gov under the access number NCT05124444.

### Eligibility criteria

Tolerant patients (i) were transplanted with an allogenic kidney; (ii) were 18 y or older; (iii) maintained good allograft function (serum creatinine < 1.7 mg/dL and proteinuria ≤ 1 g/day or /g creatinine; or values above these limits but stable with a maximum variability of 20%) in the absence of immunosuppression for at least 1 year. Patients with a history of bone marrow/other solid organ transplantation or who had been included in a protocol of tolerance induction were not eligible. Control patients were (i) healthy persons or persons with a condition unrelated to kidney or immunological disease; (ii) of European ancestry according to the investigator. All participants signed a written informed consent form approved by their local institutional review board.

### DNA samples

Following a large European survey involving 256 renal transplantation centers in 28 countries (2), we collected DNA samples and clinical data from 40 tolerant patients originating from 12 countries. Twenty-two patients' DNA samples were available at Nantes University, FR (IRB protocol number: RC14_0431) and 18 other patients were prospectively sampled as part as the TOlerance MOlecular and Genome-wide studies with Renal Allograft recipient Material [TOMOGRAM study, launched by the ERA-EDTA DESCARTES working group (http://wwwa.era-edtaworkinggroups.org/en-US/group/descartes)]. The samples originating from the Nantes University biocollection were sequenced over 100 base pairs from both ends on an Illumina HiSEq2000 (Aros Applied Biotechnology, DK) with a mean depth of 68 ± 7. The remaining 18 patients DNAs were paired-end sequenced over 125 base pairs on an Illumina HiSeq 1500 platform (Brightcore, BE) with a mean depth of 121 ± 28. The exome sequencing data from the 40 tolerant patients were compared with the dataset of 197 *in house* controls previously sequenced the same way as the TOMOGRAM cases. Details on DNA sequencing and variant analysis are available in the Supplementary material. Characteristics of tolerant patients are given in Supplementary Table 1.

### Principal component analysis

PCA of exome data is a commonly used multivariate analysis that reduces the data's dimensionality while their covariance remains preserved. This analysis reduced the high dimensional dataset to a small number of dimensions termed principal components. Here we performed a principal component decomposition of the whole set of exome data (*n* = 85,899 variants after applying the filters described above), without LD pruning, to identify outliers due to ancestry using the function snpgds PCA of the R Bioconductor package SNPRelate v1.12.2.2.

### Case-control analysis

The rarity of operational tolerance—our cohort of 40 patients is one of the world largest—(3) represents a major obstacle to the exhaustive study of its underlying genetics. However, extreme phenotypes are often driven by highly penetrant genetic variants of large effect making small (9, 13) or very small samples (16–18) sufficient to identify the causative gene. We reasoned that our limited number of patients would allow for discovering such variants, if present, and may encourage building larger cohorts in the future. To demonstrate our hypothesis, we built a pilot study comparing 40 tolerant patients to 197 controls from the general population with the objective to demonstrate enrichment of variants with large effect in the tolerant patients. This design called “*single extreme vs. control*” has proven to be an effective and reasonable method to increase the study power without resorting to a high number of, less accessible, exome-sequenced patients with the opposite phenotype (19). Healthy controls are specially relevant when we study a rare trait because the contamination in the control population will remain low with little or no impact on the study power (18). Finally, given the high number of genes/variants tested, we reported both false discovery rates (FDRs) and *p*-values following Bonferroni correction as measures of statistical significance after correction for multiple hypothesis testing (20).

In practice, we filtered exome data for moderate to high-impact variants affecting exonic, gene promoter or splice-site regions (for details see Supplementary material) and performed a principal component analysis (PCA) to identify outliers by ancestry. We studied exome-wide association using an optimal sequence kernel association test with small sample adjustment (aSKAT-O) which behaves well over a wide range of allele frequencies (21). This test can detect differences in both rare and common variants but upweights the contribution of rare variants that are likely trait-causing. The aSKAT-O can furthermore be used as a burden test or a variance component test by changing the kernel weight (Burden configuration when Rho = 1; SKAT configuration when Rho = 0) (Supplementary Table 2). We introduced principal components (PCs) 1 to 3 as covariables into our aSKAT-O model in order to correct for a possible population stratification artifact and an additional PC issued from the comparison of patients according to the sequencing machine in order to correct for a possible batch effect. We performed both by-gene analysis, with the aim to capture allelic heterogeneity, and by-variant analysis.

### Control of the variants

All genes and variants arising from aSKAT-O analyses had to pass three control steps to ensure they were true and not resulting from a batch effect due to the use of two different sequencers. Firstly, all variants were carefully examined (at the level of the BAM files) for any irregularity in the alignment processes. All variants but one (rs529437974—lack of material) were confirmed by Sanger sequencing. Secondly, a new aSKAT-O was performed with the sequencer as an outcome (instead of the phenotype) and the genes or variants predicting the sequencer were excluded (Supplementary Figure 1). Thirdly, we performed a new SKAT analysis comparing our controls (*n* = 192) to the tolerant patients after exclusion of the 9 non-European (3 Maghrebin, 6 Turkish) (*n* = 31) in order to rule out a spurious genetic association due to population stratification. Of note, three variants shared by a pair of tolerant patients each were further investigated to rule out a family relationship between the carriers that could bias the genotype-phenotype association. Patients carrying the same variants were compared for the proportion of single nucleotide polymorphisms (SNPs) with a minor allele frequency (MAF) > 5%) (Supplementary Table 2).

## Results

### Principal component analysis

**Figure 1:**
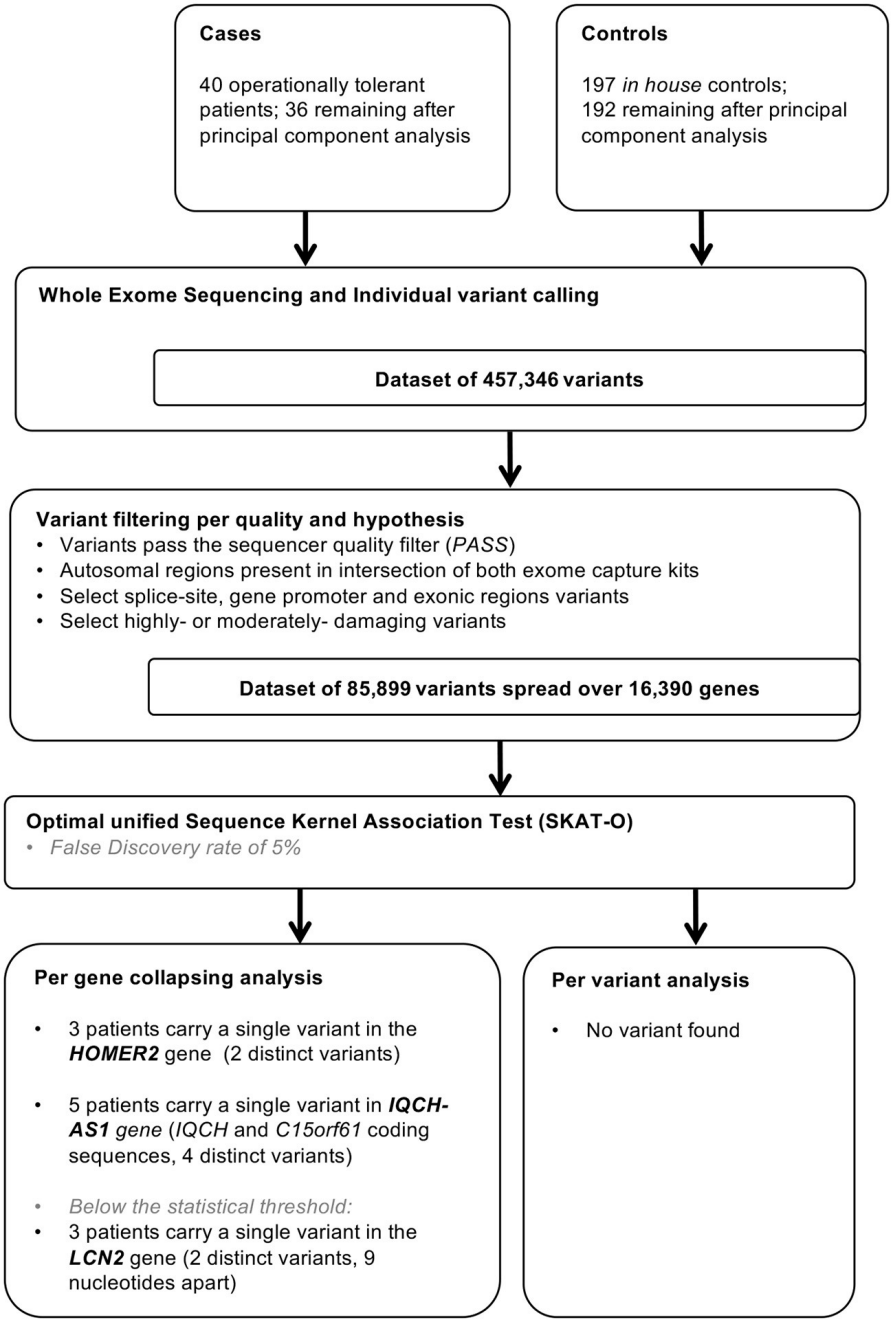
Study flow chart. In house controls were selected for absence of history of renal dysfunction and for homogeneity in principal component analysis.

**Figure 2:**
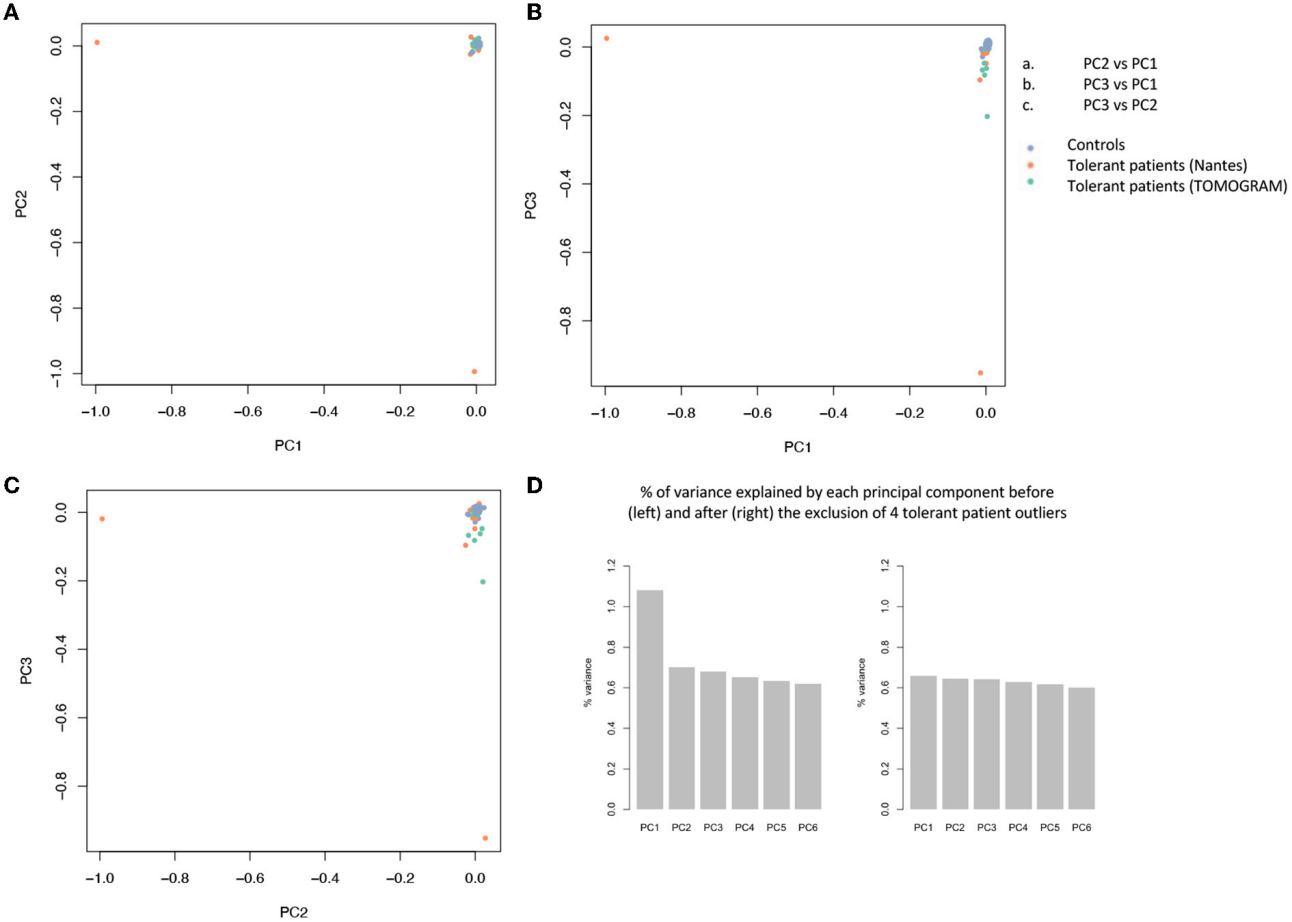
Principal component analysis (PCA) of 85.899 variants. PCA is used to reduce the dimensionality of a data set with many variables/features while minimizing the information loss and thus capturing as much of the data's variability as possible. The principal components are linear functions of the variables in the original data set. Principal components are identified by solving an eigenvalue/eigen vector problem. **(A–C)** Each dot represents a patient and is distributed in function of the 2 first principal components. Blue dots represent controls, orange dots represent tolerant patients sequenced on the AROS platform (Nantes cohort), green dots represent patients sequenced on the BIGRE platform (TOMOGRAM cohort). **(D)** % of variance explained by each principal component before (left) and after (right) the removal of 4 tolerant patient outliers (T26, T43, T53, and T63).

The filtration steps (see Study Flow Chart in Figure 1) yielded 85,899 moderate to high impact variants in 16,390 genes. PCA of exome data excluded 5 outliers by ancestry out of 197 controls and 4 (T26, T43, T53, and T63) out of 40 tolerant patients (Figure 2).

### Per gene and per variant comparisons

**Figure 3:**
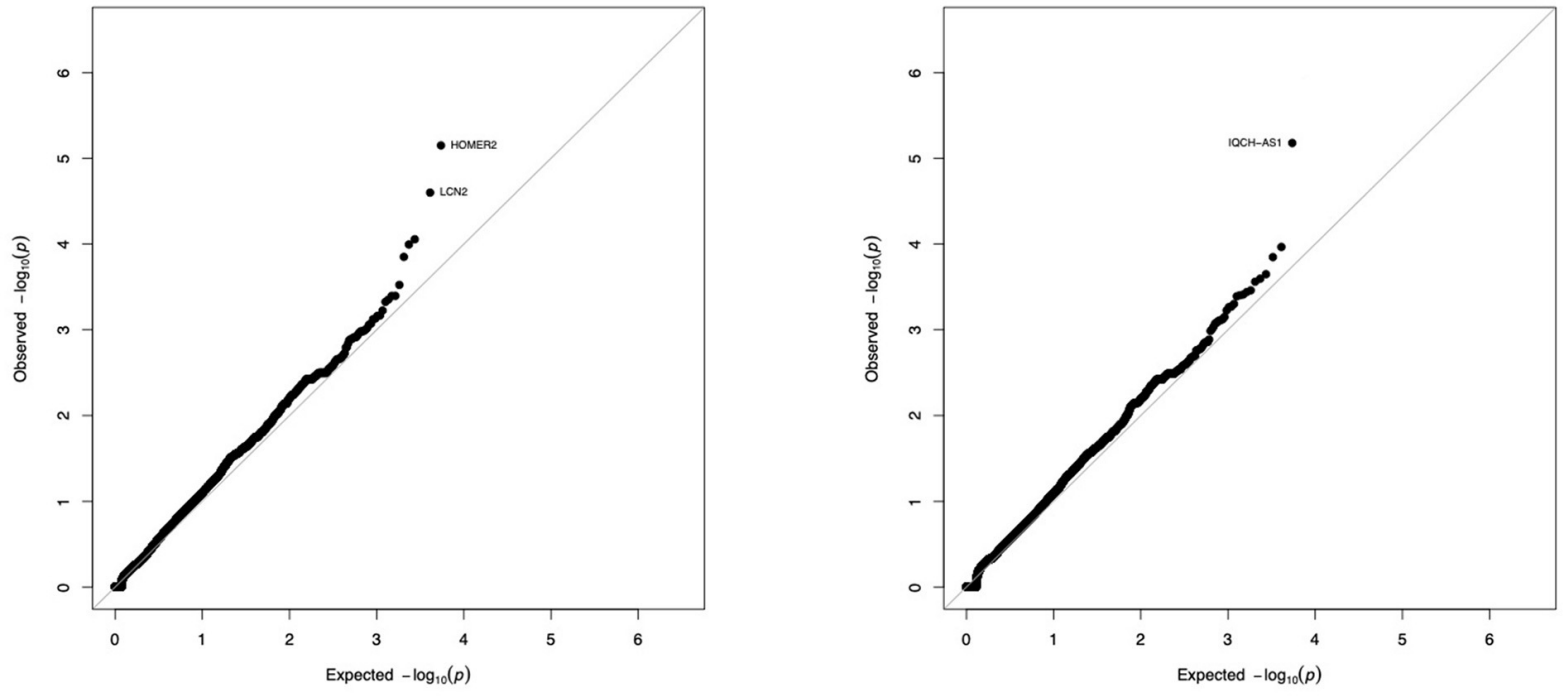
Gene-by-gene comparison of tolerant patients and controls. The distribution of exome variants with predicted moderate or high impact was compared in 36 kidney allograft tolerant recipients and 192 unrelated controls of European ancestry, using SKAT-O adjusted for small sample size. Results are presented as –log_10_ quantile-quantile plots of observed *P*-values (Y axis) vs. expected *P*-values (X axis) if variants were equally distributed in cases and controls, when Rho is set to 0 (SKAT-like test, **left panel**) or Rho is set to 1 (Burden–like test, **right panel**). Five genes (3 on the **left panel**, 2 on the **right panel**) were not considered significant because of an artefactual signal produced by the sequencing machine (see Supplementary Figure 1).

We compared the distribution of variants within each gene (*n* = 16,390) in the 36 tolerant patients vs. 192 controls (Table 1, Figure 3). We identified two genes *HOMER2* (entrez ID 9455) and *IQCH-AS1* (entrez ID 100506686); FDR = 0.0387 and 0.0362, respectively; *p* = 0.1160 and 0.1086, respectively. Three patients (8%) carried a variant in *HOMER2*. The same variant (rs79448007, p.Glu266Gly) was found in two patients (T38 and T62, Table 1). Five patients (14%) carried a *IQCH*-AS1 gene variant, two of whom shared the same variant (*p* = 0.03 for the null hypothesis of random selection among the tolerant group, Supplementary methods) (T23 and T28, Table 1). No control subject carried a moderate or highly damaging variant in these two genes. *IQCH-AS1* encodes a long non-coding RNA, and variants were filtered in because the complementary DNA strand encompasses two protein-coding genes: *IQCH* (most of IQCH-AS1 length) and *C15orf61*. These two genes remain significant after excluding the 9 tolerant patients of foreign origin (data not shown). A third gene, *LCN2* (entrez ID 3934) showed a weaker association (FDR = 0.102; *p* = 0.4847), with three patients (8%) carrying a variant vs. 0 controls. Again, two patients shared the same *LCN2* variant (T38 and T54, Table 1). Both *LCN2* variants were located 9 base pairs apart, in the 20 amino-acid long signal peptide of the encoded NGAL protein, suggesting the possibility of a shared functional effect. Of note, one patient (T38) carried both a *LCN2* and a *HOMER2* variant. The 8 variants identified in the 10 patients were rare [i.e., allele frequency < 1% (22)] single nucleotide substitutions. All three genes encode proteins that affect primary cilia function (*p* = 0.01; Supplementary methods). We ruled out close ancestry in patients sharing a same variant by quantifying their SNPs homology (Supplementary Table 3). The clinical characteristics of the tolerant patients with genetic variants are given in Table 2. Four out of the 10 tolerant patients had received a fully-matched graft for *loci* HLA A, B and DR. Interestingly, patient T38 who carried variants in both *HOMER2* and *LCN2* had remained tolerant over an especially long time (173 months at the time of this analysis). SNP array analysis in a subgroup of 19 tolerant patients (from Nantes) detected no copy number variants (CNVs) over any of the three loci (Supplementary methods).

**Table 1:** Characteristics of the variants associated with tolerance through per-gene aSKAT-O.

**Variant**	**Nb of reads with the change/wild allele/another change**	**Gene**	**Variant ID**	**Protein**	**Predicted consequence**	**CADD score**	**MAF (All/non-Finish European)**	**Controls**	**Cases**	**FDR**
chr15:83,518,616_G/A	*78/71/0*	*HOMER2*	rs529437974	Homer-2	Missense variant R306C	27.5	5.2e-5/8.8e-5	0	T3	0.0386
chr15:83,519,982_T/C	*32/30/ 1*	*HOMER2*	rs79448007	Homer-2	Missense variant E266G	33	0.006/0.008	0	T38	
	*16/23/1*								T62	
chr15:67,713,634_G/A	*28/28/0*	*IQCH-AS1*	rs151170401	IQCH	Missense (new START codon) V742M	22.3	0.0002/3.6e-5	0	T46	0.0362
chr15:67,757,607_C/T	*51/41/0*	*IQCH-AS1*	rs36067711	IQCH	Missense variant T883I	0.002	0.003/0.001	0	T23	
	*43/56/1*								T28	
chr15:67,786,628_T/G	*19/11/0*	*IQCH-AS1*	rs569975447	IQCH	Missense variant M965R	22.9	8e-6/1.8e-5	0	T9	
chr15:67,813,744_T/C	*33/34/0*	*IQCH-AS1*	rs187386601	C15orf61	Missense variant V53A	23.2	0.0003/ 8.7e-5	0	T52	
chr9:130911821_T/C	*92/74/0*	*LCN2*	rs139418967	NGAL	Missense variant L6P	22.8	0.001/0.002	0	T38	0.1019007
	*55/71/0*								T54	
chr9:130,911,830_G/T	*74/74/0*	*LCN2*	rs147787222	NGAL	Missense variant G9V	19.73	0.001/0.002	0	T42	

Variant IDs are given according to dbSNP155, MAF are given according to GnomAD exomes values for all patients/ non-Finish European only (on 13th April 2021); ID designates Tolerant patient ID number and conform with our numbering in Massart, Pallier et al. in Nephrol Dial Transplant 2016. All variants found were heterozygous. Three variants from this list were found in a couple of patients.

MAF, Minor Allele Frequency; Nb, number.

**Table 2:** Characteristics of the variant-bearing tolerant patients identified through per-gene aSKAT-O.

**ID**	**Sex and ethnicity**	**Primary nephropathy**	**Age at transplantation (years)**	**HLA A-B-DR mismatches**	**Initial IS**	**Cancer history during IS period**	**Reason for IS withdrawal**	**Age when becoming tolerant (approximate, in years)**	**Tolerance duration (months)**	**Dialyse-free period (months)**	**Tolerance interruption (cause)**	**Gene variants (rs)**
T03	M, Caucasian	Autosomal dominant polycystic kidney disease	18	0-1-1	FK, AZA, steroids	no	Non-compliance	21	14	66	Worsening function, no biopsy	*HOMER2* (rs529437974)
T09	M, Caucasian	unknown	30	0-0-0	IL2RA, CsA, MMF, steroids	no	Meningo-encephalitis	38	0	77	NA	*IQCH-AS1* (rs569975447)
T23	M, Caucasian	Membranoproliferative glomerulonephritis	43	0-0-0	AZA, steroids	Multiple skin squamous cell carcinoma	Non-compliance	60	274	274	NA	*IQCH-AS1* (rs36067711)
T28	F, Turkish	Chronic pyelonephritis secondary to vesico-ureteral reflux	24	0-0-0	CsA, AZA, steroids	lymphoma	Non-compliance	40	49	64	Worsening function, no biopsy	*IQCH-AS1* (rs36067711)
T38	M, Caucasian	Undetermined glomerulopathy	41	1-2-1	ATG, CsA, AZA, steroids	0	Non-compliance	54	173	173	NA	*HOMER2* (rs79448007)
												*LNC2*
												(rs139418967)
T42	F, Caucasian	Congenital renal hypoplasia	27	2-1-1	ATG, CsA, AZA, steroids	0	Non-compliance	36	18	46	Humoral rejection	*LNC2* (rs147787222)
T46	M, Caucasian	IgA nephropathy	54	0-1-1	AZA, steroids	Recurrent skin squamous cell carcinoma	Cancer and non-compliance	67	181	181	NA	*IQCH-AS1 (rs151170401)*
T52	M, Caucasian	Focal segmental glomerulosclerosis	63	1-1-1	ATG, CsA, AZA, steroids	no	Non-compliance	76	94	101	Worsening function, no biopsy	*IQCH-AS1* (rs187386601)
T54	F, Caucasian	Congenital renal hypoplasia	34	2-2-0	ATG, CsA, AZA, steroids	Lymphoma	Cancer	51	80	80	NA	*LNC2* (rs139418967)
T62	F, Caucasian	Autosomal dominant polycystic kidney disease	32	0-0-0	IL2RA, FK, MMF, steroids	no	Non-compliance	37	73	73	NA	*HOMER2* (rs79448007)

Tolerance duration refers to the total period with a normal kidney allograft function (see methods) in the absence of immunosuppression while dialyse-free period refers to the total period with a functioning kidney allograft irrespective of creatinine or proteinuria levels.

TAG, anti-thymocyte globulin; AZA, azathioprine; CAAD, combined annotation-dependent depletion; CsA, cyclosporin; FK, FK-506 binding protein or tacrolimus; ID, patient identity; IL2RA, interleukine-2 receptor antagonist; IS: immunosuppression; MAF, minor allele frequency; MMF, mycophenolate mofetil; NA, not applicable; rs, dbSNP reference number.

We then used the aSKAT-O to compare the frequency of each variant (*n* = 85,899) in the 36 tolerant vs. 192 controls, and did not identify any variant with statistical significance.

## Discussion

Overall, our results show a statistically significant association of spontaneous renal tolerance with moderate to high impact coding variants in *HOMER2* and *IQCH/C15orf61*, and a likely association with *LCN2*. HOMER2 is an actin-binding, scaffolding protein expressed at various specialized primary cilia (23), and a critical regulator of intracellular Ca^2+^ signaling (24). Homer2 and 3 compete with calcineurin, resulting in a negative regulation of T-cell activation and Homer2,3-deficient mice develop an autoimmune-like pathology (25). *IQCH* encodes the IQ motif-containing protein H also known as SPATA17 (spermatogenesis associated 17), a cilia and flagella-associated protein involved in spermatogenesis (26). IQ motif containing proteins bind calmodulin (27), the key transducer of intracellular calcium signals in T cells, and alter its binding to calcium. *C15orf61* (chromosome 15 open reading frame 61) is an uncharacterized coding gene located immediately downstream of *IQCH* in a non-imprinted portion of chromosome 15. *LCN2* is expressed at the primary cilium (28, 29). It encodes NGAL (neutrophil gelatinase-associated lipocalin), an iron-trafficking protein involved in multiple immunological processes (30–32), including common variable immunodeficiency (33), but its role in transplantation remains obscure (34, 35). Furthermore, HOMER2, IQCH, and NGAL are all cilia-associated proteins. The T cell cilium is viewed as a specialized compartment that assembles at the plasma membrane upon TCR binding and interaction with the antigen-presenting cell or target cell, producing the so-called “immune synapse” (36, 37). The immune synapse is a place of intense TCR/CD3 recycling which directly impacts on the duration and intensity of TCR signaling. This process is dependent upon actin polymerization and intracellular calcium release and is critical for the development of T cell full effector potential (38, 39). A spurious association between renal tolerance and the primary cilium resulting from a cluster of ciliopathies in our cohort appears unlikely. While 2/10 variant-carrying tolerant patients suffered from a recognized ciliopathy (Table 2), none of the genes above are associated with a renal disease, either in the OMIM database or in a large whole-exome study (40). Furthermore, our results are consistent with the reported association of tolerance with a coding variant in *PARVG*, encoding an actin-binding protein (41), and also with a GWAS of the mirror phenotype, acute rejection of renal allograft, that showed a signal close to the *CCDC67* locus (42), encoding a key protein for ciliogenesis (43). Finally, accumulating reports of immunological disorders linked to dysfunctional actin dynamics at the immune synapse further support the causality of the association (44, 45). It might seem contradictory that patients carrying putative tolerance variants would still be immunocompetent. It is however well-known that transplantation tolerance in primates cannot be achieved by blocking a single mechanism (46, 47). It is therefore likely that renal allograft tolerance results from a combination of the hereditary predisposition that we showed here, and the use of multiple immunosuppressive drugs at the time of transplantation. To our knowledge, this is the first unbiased, exome-wide study of tolerance in solid organ transplantation. While we identified genetic variants in only a subset of our patients (10/36, 27%), indicating causal heterogeneity, other mechanisms, e.g., epigenetic changes, might possibly involve the same molecules, or other molecules in the same pathway, like Homer1 as suggested elsewhere (48). The unusually high proportion (25%) of donor-recipient pairs that were fully matched for the HLA A, B and DR *loci*, also argues for a possible contribution of donor-recipient genetic interactions (49).

Our study has several limitations. First, because documented cases of allograft tolerance in renal transplantation are extremely rare, we could not replicate our results in an independent cohort. Our study stems from a near-complete survey of more than 218,000 renal transplant recipients in 28 countries, where tolerance was reported in 3 per 10,000 renal recipients (2). Contrasting with other successful genetic projects on common phenotypes (diabetes, chronic kidney disease, and many more), our recruitment was necessarily limited. We designed our study specifically to overcome this issue (SKAT adjusted for small samples, FDR, enlarged control group), resulting in several significant rare variants in biologically relevant genes. While a plethora of GWA studies for many kinds of traits has called for stringent guidelines on *p*-values and replication cohorts, the number of tolerant patients needed to meet these standards is simply not feasible. Nevertheless, the FDRs of ~0.03 are highly convincing, indicating only a 3% chance that the results are false discoveries (20). Second, while our study allowed to unmask a few cases of possibly Mendelian tolerance—akin to studies of rare Mendelian diseases where only a few patients can uncover the underlying gene (16, 17)—it was underpowered to detect heterogeneous and/or polygenic factors in the remaining patients. We may also have missed variants of large effect as we did not cover every exon of the genome, partly because of the limits of exome capture kits (50). Finally, in the context of a rare trait and *a posteriori* identification we must acknowledge that data collection could not be exhaustively controlled. Nevertheless, this is the first exome-wide study of tolerance and provides a springboard for candidate gene studies and meta-analyses.

In conclusion, our work strongly suggests that spontaneous renal allograft tolerance stems from moderate to high impact, coding variants of primary cilium genes in at least a sizeable subgroup of patients (10/36, 27%). Further research should confirm our results and investigate the mechanisms involved.

## Data availability statement

The bam files of the 38 tolerant patients who consented to further use of their data by all bona fide researchers are deposited in the European Genome-Phenome Archive (EGAS00001007154 and 155).

## Ethics statement

The studies involving human participants were reviewed and approved by Erasme-ULB Ethics Committee, Université Libre de Bruxelles, Brussels, Belgium. The patients/participants provided their written informed consent to participate in this study.

## Author contributions

DA and MA conceived the study. AM and OV implemented the biocollection of the TOMOGRAM study with the support of the DESCARTES ERA-EDTA board members and MG the biocollection of Nantes University with the support of the DIVAT Consortium. AM, OV, MG, MS, JP, MM, and the RENAL TOLERANCE INVESTIGATORS managed IRB approval process and collected patient data and samples. AM and MG reviewed patients' phenotypes. JD, RBS, ET, SVD, and CV obtained IRB-approved and consent from control patients. AM, CP, and VJ performed DNA extraction, preparation of the samples, and Sanger validation of the variants. BC, DC, and DD created patient libraries, sequenced the samples, and prepared them for CNV analysis. JS performed variant calling and annotation. LG, JS, DC, GS, SD, VJ, IP, OV, and PH contributed significant intellectual content. CO performed the principal component analysis and the case-control study under the guidance of ME. AM, CO, RD, ME, DA, SB, and MA analyzed the data and wrote the manuscript. AM, MA, SB, and DA supervised all aspects of the project. All authors revised the manuscript. All authors contributed to the article and approved the submitted version.
