# Erratum: An exome-wide study of renal operational tolerance

**DOI:** 10.3389/fmed.2023.1235101

**Published:** 2023-06-20

**Authors:** Frontiers Production Office

**Affiliations:** Frontiers Media SA, Lausanne, Switzerland

**Keywords:** exome sequencing, renal transplantation, operational tolerance, NGAL, *LCN2*, *Homer2*, *IQCH*, primary cilium

Due to a production error, the consortium Renal Tolerance Investigators was not correctly listed in the article.

The section **Group members of the Renal Tolerance Investigators** now appears at the end of the article.

The publisher apologizes for this mistake. The original article has been updated.

