# Supplementary material for: An exome-wide study of renal operational tolerance
Source: Front Med (Lausanne). 2023 May 17;9:976248. doi: 10.3389/fmed.2022.976248 (PMC10230038; doi:10.3389/fmed.2022.976248)
Supplement: Supplementary file 1 [file Data_Sheet_1.pdf]

Supplementary Material

Table of content

SUPPLEMENTARY METHODS 2

DNA extraction and exome sequencing 2

Read mapping, variant filtering and variant analysis 2

Copy number variations 3

Calculation of p-values 3

SUPPLEMENTARY TABLES 5

S-Table 1. Reported characteristics of tolerant patients. 5

S-Table 2. Settings of by-gene aSKAT-O and identified genes 7

S-Table 3. Homology of common SNPs in pairs of tolerant patients carrying a same rare variant associated with tolerance. 8

S-Table 4. Merging the two BED files issued from the two exome capture kits used 9

SUPPLEMENTARY FIGURE 10

S-Figure 1. Crossed SKATs of sequencing platform versus phenotype. 10

SUPPLEMENTARY REFERENCES 11

SUPPLEMENTARY METHODS

DNA extraction and exome sequencing

The 22 DNA samples from the Nantes biocollection were extracted as described previously;[1] DNA libraries were prepared using the TruSeq DNA LT Sample Prep Kit (Illumina Inc., US-CA), according to manufacturer's instructions. Briefly, one microgram of DNA underwent acoustic fragmentation (Covaris Inc., US-MA), end-repair, adenylation, adapter ligation and amplification by polymerase chain reaction (PCR). The resulting shotgun libraires were captured for exome enrichment with the TruSeq exome enrichment kit v1.2 (Illumina Inc.). The exomes (45 Mb) were sequenced over 100 base pairs from both ends (pair-ended) on an Illumina HiSEq2000 (Aros Applied Biotechnology, DK) yielding an average of 124±43 millions paired-end reads per patient with a mean depth of 68±7. The remaining 18 DNAs originating from the TOMOGRAM Study were extracted manually from whole blood for the purpose of the present study, by an anion-exchange resin method (Qiagen genomic DNA purification kit, DE). DNA concentrations were assessed by spectrophotometry (Nanodrop Instrument, Thermo Fischer, US-MA). One microgram of DNA underwent acoustic fragmentation (Covaris Inc.) and entered the library preparation process with the KAPA HyperPrep kit (Roche Inc., CH). The exome sequences (64 Mb) were captured using SeqCap EZ Human Exome v3.0 (Roche Inc.) and pair-ended sequenced over 125 base pairs on an Illumina HiSeq 1500 platform (Brightcore, BE) yielding an average of 129±32 millions paired-end reads per patient with a mean depth of 121±28. The 197 local controls were sequenced previously using the same method as the 18 TOMOGRAM cases.

Read mapping, variant filtering and variant analysis

All patient data were processed individually with the same bioinformatic pipeline as follows. Raw data were aligned to human genome build 37 (hg19) with the Burrows-Wheeler Alignment Tool using BWA-MEM 0.7.12).[2] Picard Markduplicates tools 2.9.0 was used to mark duplicate reads. The Genome Analysis ToolKit Haplotype Caller 3.3 was used for local realignment around indels, base quality score

recalibration and single nucleotide polymorphism (SNP) an indel calling according to best practice recommendations[3]. Variants were called using Haplotye Caller.[4] The variants that did not pass the 'PASS' platform quality filter according to GATK's hard filtering best practice recommendations (QD < 2.0; ReadPosRankSum < -8.0; FS > 60.0; MQRankSum < -12.5; MQ < 40.0) were rejected. Then, based on our clinical hypothesis, we selectively retained moderate to high-impact variants affecting exonic, gene promoters or splice-site regions across the autosomes using the Bioconductor package Variant annotation 1.24.5.[5] Considering the two different exome capture kits, we restricted our analysis to the exonic sequences targeted by both kits using Bedtools (v2.27.0) (Supplementary Table 4). For each variant we used the rs number reference, when available, according to dbSNP155 and provided an estimation of pathogenicity using the Combined Annotation-Dependent Depletion (CADD) score.[6] Principal component analysis (PCA) of exome data was performed to identify outliers by ancestry using the R package SNPRelate v1.12.2.

Copy number variations

Nineteen patients from the Nantes cohort were tested for copy number variants (CNVs) overlapping the loci of the variants identified in this study, using a Single Nucleotide Polymorphism array approach (CytoSNP-12, Infinium HD, Illumina) as recommended by the manufacturer. Results were read-out on the CNV Webstore site[7].

Calculation of p-values

To calculate a p-value for the probability that all three genes are "cilia genes," we condition on the number of genes found (three) and the fact that the first is a cilia gene. Then, the probability that the next two are cilia genes is $(2{,}000/16{,}390)^2 = 0.01$, 2,000 being a upper estimate of the total number of cilia genes in the genome[8] and 16,390 being the total number of genes tested. This assumes equal and random selection of genes. Hence, the null hypothesis of equal, random probabilities of finding three such genes is rejected. To find the p-value for the event that two people carry rs36067711, we

condition on the fact that at least one person is a carrier. Then, the probability that none of the other 35 is a carrier equals $(1-0.001)^{35}$, where 0.001 is the MAF for the variant. The probability that at least one other person is a carrier is $1-(1-0.001)^{35}=0.03$, under the assumptions of equal random chances of anybody being a carrier. Hence, we again reject the null hypothesis of equal random chances (0.001) that tolerant patients are carriers of this variant.

SUPPLEMENTARY TABLES

S-Table 1. Reported characteristics of tolerant patients.

Parameters	Patients (N= 40)
- tolerant (optimal function*) (no.)<br>- tolerant (suboptimal stable function**) (no.)	38<br>2
Male sex	28/40
Ethnicity (no.)<br>- European<br>- Northern African<br>- Turkish	 31<br>3<br>6
Age at transplantation (years)	33±16
Cold ischemia duration (minutes)	1262 [780-1627]
Age at complete cessation of immunosuppression (years)	43±15
Circumstances of immunosuppression cessation (no.)<br>- noncompliance<br>- cancer or severe infection<br>- pregnancy<br>- poor graft function	 31<br>7<br>1<br>1
Donor age (yr)	30±13
Donor male sex (%)	11/36
Donor status (no.)<br>- deceased<br>- living	 29<br>11

Full matched recipients (no.)	10/40
HLA mismatches in mismatched recipient (no.)<br>- A<br>- B<br>- DR<br>- A-B-DR	 1±0.6 (n=29)<br>1.2±0.6 (n=29)<br>0.8±0.7 (n=27)<br>3±1 (n=27)
Immunisation (no.)<br>- positive PRA before transplantation<br>- positive PRA at the time of transplantation<br>- pretransplantation donor-specific antibodies<br>- De novo donor specific antibodies	 7/28<br>2/21<br>1/25<br>8/33
Duration of tolerance (months)	96 [50-142] (n= 38)
Time interval without dialysis nor immunosuppressive drugs (months)	115 [68-156] (n=40)
Last serum creatinine while tolerant (mg/dl)	1.2±0.3
Tolerant patients with proteinuria (300-1000 mg/day) (no.)	31/38

Note: results are expressed as a number, or mean ± standard deviation, or median and interquartile range. An optimal function (*) designates a serum creatinine <1.7 mg/dL and proteinuria ≤1g per day or per g creatinine during at least one year without immunosuppression while a suboptimal function (**) designates an allograft function beyond these criteria but with stable function with less than 20% variation over at least 1 year. The number of patients involved in each analysis is specified under brackets only when it differs from the rest of the column.

Abbreviations: no., number; PRA, Panel Reactive Antibody. A positive PRA was defined as a non-null PRA.

S-Table 2. Settings of by-gene aSKAT-O and identified genes.

		Gene name		
	Rho	*HOMER2*	*IQCH-AS1*	*LCN2 (*)*
36 tolerant *vs* 192 controls	0	X	-	X
	1	-	X	-

Note: Rho = 1 corresponds to a burden-like configuration while a Rho = 0 corresponds to a SKAT configuration of the aSKAT-O. Burden tests allow to assess the cumulative effects of multiple variants in a genomic bin. They are especially powerful when a large proportion of variants in a region are rare and causal, i.e. influence the phenotype in the same direction. SKAT tests are variance component score test. They become more powerful than burden tests when a genetic region has both protective and deleterious variants or many noncausal variants.[9; 10]

(*) The data regarding *LCN2* show the setting where this gene almost reached statistical significance (FDR=0.102)

S-Table 3. Homology of common SNPs in pairs of tolerant patients carrying a same rare variant associated with tolerance.

Three pairs of tolerant patients shared a single specific variant (T38 and T62 both carried rs7944807 (in *HOMER2*), T23 and T28 both carried rs 36067711 (in *IQCH*) while T38 and T54 both carried rs139418967 (in *LCN2*). We ruled out a close relatedness (identity by descent) between patients by examining the fraction of SNPs they shared using Highlander (https://sites.uclouvain.be/highlander/). With homology ranging from 48 to 52%, a close relatedness seems unlikely.

	T23	T28	T38	T54	T62
T23	100% (7 629)	**52% (10 015)**	49% (9 979)	48% (10 029)	52% (10 061)
T28		100% (7 554)	48% (9 962)	49% (9 917)	52% (10 030)
T38			100% (7 227)	**51% (9 537)**	**48% (10 055)**
T54				100% (7 189)	49% (10 011)
T62					100% (7 678)

Method: when comparing 2 samples S1 and S2, all variants from S1 and S2 having a dbSNP identity, an allele frequency in gnomAD (exomes) > 5% and a read depth > 10 were retained. The total number (in parentheses) is the sum of SNPs unique to S1, unique to S2 and common in S1 and S2. The percentage reflects the number of SNPs common to S1 and S2.

S-Table 4. Merging the two BED files issued from the two exome capture kits used.

The following BED files were merged with Bedtools v2.27.0 (on the 19 January 2018) http://bedtools.readthedocs.io/en/latest/content/tools/merge.html

1) BED File Roche SeqCap EZ Human Exome v3.0 (corresponding to the exon capture kit used at Brightcore, BE) and available here: http://sequencing.roche.com/en/products-solutions/by-category/target-enrichment/hybridization/seqcap-ez-exome-utr-kit.html
2) BED Files True seq: v1.2 (corresponding to the exon capture kit used at Arosab, DK) and available here: https://support.illumina.com/downloads/truseq-exome-product-files.html

SUPPLEMENTARY FIGURE

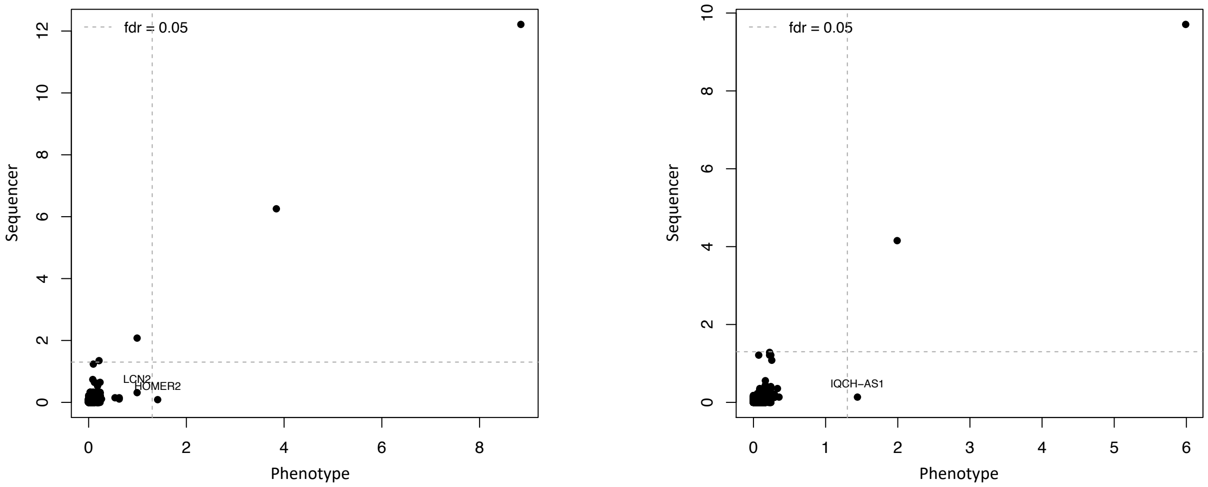

S-Figure 1. Crossed SKATs of sequencing platform versus phenotype.

The distribution of exome variants with predicted moderate or high impact was compared in 36 kidney allograft tolerant recipients and 192 unrelated controls of European ancestry, using SKAT-O adjusted for small sample size. Results are presented as FDR values of SKAT-O analyses of variant distributions in study subjects according to the exome sequencing platform used in this study (sequencer A versus B, Y axis) or according to the tolerance status (tolerant recipient versus control, X axis), when Rho is set to 0 (SKAT-like test, left panel) or Rho is set to 1 (Burden-like test, right panel). Dotted lines are set at significance level after correction for multiple hypotheses using a Benjamini FDR of 0.05. True signals are located in lower right quadrants. The unlabeled genes in upper quadrants represent sequencer-associated artifactual signals and were not reported.

SUPPLEMENTARY REFERENCES

[1] R. Danger, E. Thervet, M.L. Grisoni, P.L. Puig, A. Pallier, D. Tregouet, D. Lecorre, M. Giral, C. Legendre, J.P. Soulillou, and S. Brouard, PARVG gene polymorphism and operational renal allograft tolerance. Transplant Proc 44 (2012) 2845-8.
[2] H. Li, and R. Durbin, Fast and accurate short read alignment with Burrows-Wheeler transform. Bioinformatics 25 (2009) 1754-60.
[3] G.A. Van der Auwera, M.O. Carneiro, C. Hartl, R. Poplin, G. Del Angel, A. Levy-Moonshine, T. Jordan, K. Shakir, D. Roazen, J. Thibault, E. Banks, K.V. Garimella, D. Altshuler, S. Gabriel, and M.A. DePristo, From FastQ data to high confidence variant calls: the Genome Analysis Toolkit best practices pipeline. Curr Protoc Bioinformatics 43 (2013) 11 10 1-33.
[4] A. McKenna, M. Hanna, E. Banks, A. Sivachenko, K. Cibulskis, A. Kernytsky, K. Garimella, D. Altshuler, S. Gabriel, M. Daly, and M.A. DePristo, The Genome Analysis Toolkit: a MapReduce framework for analyzing next-generation DNA sequencing data. Genome Res 20 (2010) 1297-303.
[5] V. Obenchain, M. Lawrence, V. Carey, S. Gogarten, P. Shannon, and M. Morgan, VariantAnnotation: a Bioconductor package for exploration and annotation of genetic variants. Bioinformatics 30 (2014) 2076-8.
[6] M. Kircher, D.M. Witten, P. Jain, B.J. O'Roak, G.M. Cooper, and J. Shendure, A general framework for estimating the relative pathogenicity of human genetic variants. Nature genetics 46 (2014) 310-5.
[7] G. Vandeweyer, E. Reyniers, W. Wuyts, L. Rooms, and R.F. Kooy, CNV-WebStore: online CNV analysis, storage and interpretation. BMC Bioinformatics 12 (2011) 4.
[8] T.J.P. van Dam, J. Kennedy, R. van der Lee, E. de Vrieze, K.A. Wunderlich, S. Rix, G.W. Dougherty, N.J. Lambacher, C. Li, V.L. Jensen, M.R. Leroux, R. Hjeij, N. Horn, Y. Texier, Y. Wissinger, J. van Reeuwijk, G. Wheway, B. Knapp, J.F. Scheel, B. Franco, D.A. Mans, E. van Wijk, F. Kepes, G.G. Slaats, G. Toedt, H. Kremer, H. Omran, K. Szymanska, K. Koutroumpas, M. Ueffing, T.T. Nguyen, S.J.F. Letteboer, M.M. Oud, S.E.C. van Beersum, M. Schmidts, P.L. Beales, Q. Lu, R.H. Giles, R. Szklarczyk, R.B. Russell, T.J. Gibson, C.A. Johnson, O.E. Blacque, U. Wolfrum, K. Boldt, R. Roepman, V. Hernandez-Hernandez, and M.A. Huynen, CiliaCarta: An integrated and validated compendium of ciliary genes. PLoS One 14 (2019) e0216705.
[9] M.C. Wu, S. Lee, T. Cai, Y. Li, M. Boehnke, and X. Lin, Rare-variant association testing for sequencing data with the sequence kernel association test. Am J Hum Genet 89 (2011) 82-93.
[10] S. Lee, M.J. Emond, M.J. Bamshad, K.C. Barnes, M.J. Rieder, D.A. Nickerson, N.G.E.S.P.-E.L.P. Team, D.C. Christiani, M.M. Wurfel, and X. Lin, Optimal unified approach for rare-variant association testing with application to small-sample case-control whole-exome sequencing studies. Am J Hum Genet 91 (2012) 224-37.
